# SARS-CoV-2 genomic surveillance using rapid point of care COVID-19 antigen tests at public test sites in California

**DOI:** 10.3389/fpubh.2025.1620651

**Published:** 2025-07-25

**Authors:** Eric M. Foote, Ellen L. Bouchard, Charlotte B. Acharya, Elizabeth F. Baylis, John M. Bell, Christina Morales, Phacharee Arunleung, Andreina Urrutia Gonzalez, Jacob Conston, Stephenie Liu, Ryan Davis, Jeremy Brown, Natalie Hnyp, Siranoosh Ashtari, Alyssa Laxamana, Vanessa Rashbrook, Lutz Froenicke, Kathleen Jacobson, Richard Michelmore, Debra A. Wadford

**Affiliations:** ^1^California Department of Public Health, Richmond, CA, United States; ^2^Stanford University School of Medicine, Stanford, CA, United States; ^3^University of California, Davis, Davis, CA, United States; ^4^University of California-Davis School of Medicine, Davis, CA, United States

**Keywords:** SARS-CoV-2, COVID-19 rapid antigen test, whole genome sequencing, population viral genomic epidemiology, genomic surveillance

## Abstract

California’s SARS-CoV-2 genomic surveillance program (California COVIDNet) developed whole genomic sequencing (WGS) capability from positive COVID-19 antigen tests to maintain genomic surveillance from public test sites. Over 4-months, COVIDNet sourced specimens from positive COVID-19 antigen tests from 142 California public test sites in 43 counties. Successful WGS was defined as at least 83% reference coverage with a minimum 20x genomic read depth. There were 14,088 SARS-CoV-2 genomes obtained from positive antigen tests with a success rate of 92.9%. The program generated 13.9% of SARS-CoV-2 sequences in California and 2.7% of sequences in the US during the program operation period. In one rural region, 69% of all SARS-CoV-2 sequences were generated by the program. California successfully transitioned SARS-CoV-2 WGS on a statewide scale to specimens sourced from positive antigen tests. Community-based testing coupled with a comprehensive genomic surveillance program provides a statewide strategy applicable to other pathogens of public health significance.

## Introduction

The COVID-19 pandemic created an imminent worldwide need for genomic surveillance to identify variants of SARS-CoV-2 to inform and improve the health system response. The California Department of Public Health (CDPH) COVID-19 Testing Taskforce rapidly established free, public COVID-19 diagnostic test sites throughout California to meet the need for COVID-19 testing and SARS-CoV-2 genomic surveillance, designated as California COVIDNet (1). Residual positive SARS-CoV-2 specimens were sourced for genomic surveillance, via whole genome sequencing (WGS), to characterize and monitor the SARS-CoV-2 variants within California, ensuring representation across the state, including rural and remote regions (1, 2).
Rapid, point of care, lateral flow SARS-CoV-2 (COVID-19) antigen tests first became available in September 2020 (3, 4). COVID-19 rapid antigen tests provided results in 15 min, were low cost, and could be performed at the point of care (3). This improved testing access, especially in rural areas, and enabled immediate isolation of individuals with COVID-19 as well as immediate access to treatment, meeting public health and patient care needs. California COVID-19 public test sites adopted rapid COVID-19 antigen tests as the primary form of testing in May 2022. However, the change from molecular-based laboratory performed tests to point of care antigen tests at California’s public COVID-19 test sites reduced the availability of SARS-CoV-2 specimens for California’s genomic surveillance program. At the time, large-scale population-based genomic surveillance was conducted from molecular-based tests (5). Due to CDPH’s successful SARS-CoV-2 WGS from positive COVID-19 antigen tests during the California Arriving International Traveler COVID-19 testing program that began in December 2021 (manuscript in preparation), we extended statewide SARS-CoV-2 genomic surveillance to include positive COVID-19 rapid antigen tests for WGS (1, 2). Here we report the SARS-CoV-2 genomic surveillance results from positive COVID-19 antigen tests from public test sites leveraging the California COVIDNet infrastructure.

## Methods

### CDPH sponsored COVID-19 test sites

**Figure 1:**
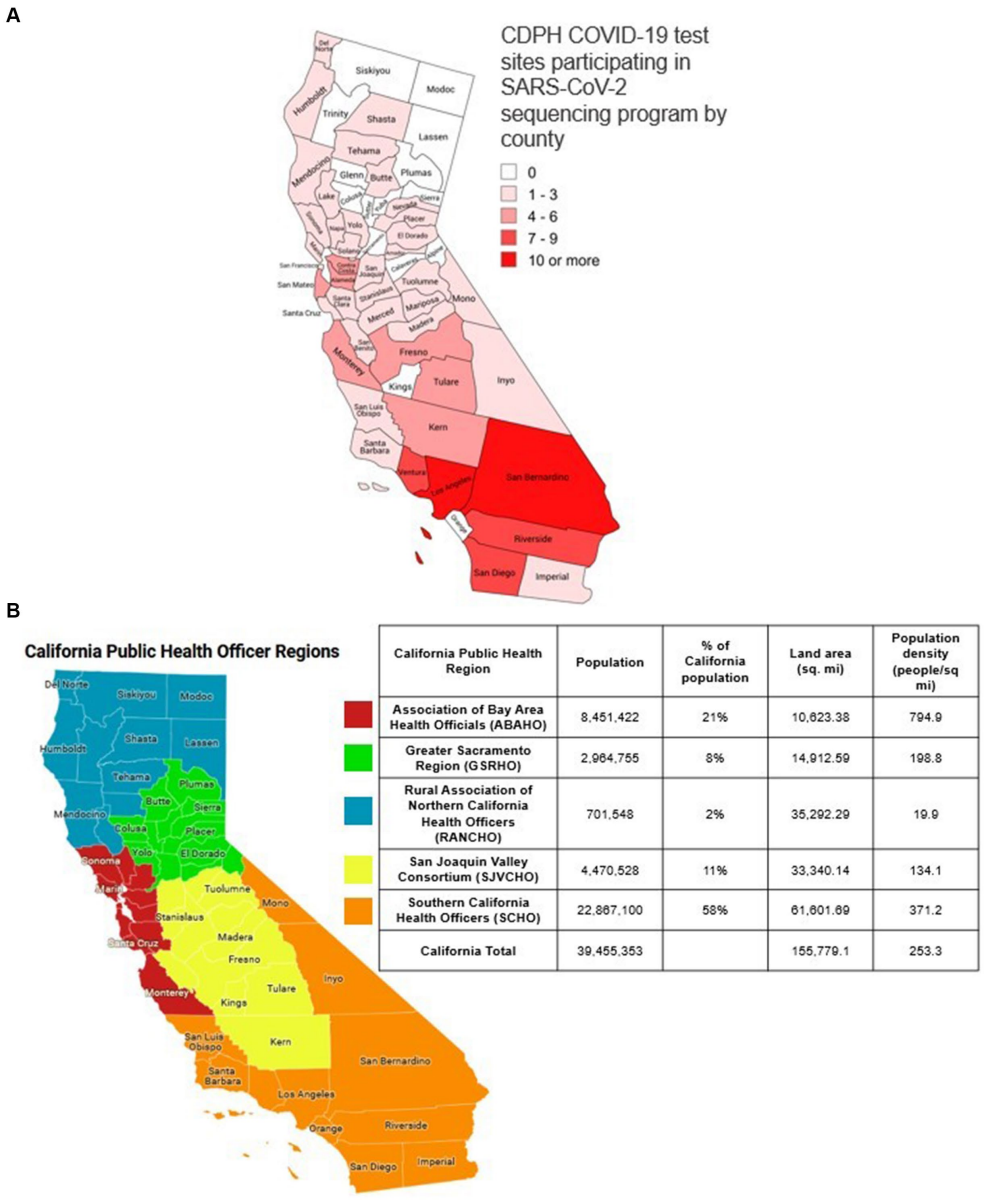
**(a)** Map of the public health officer regions in California. The five California public health officers regions. Red: ASSOCIATION of Bay Area Health Officials (ABAHO); Green: Greater Sacramento Region of Health Officers (GSRHO); Blue: Rural Association of Northern California Health Officers (RANCHO); Yellow: San Joaquin Valley Consortium of Health Officers (SJVCHO); Orange: Southern California Health Officers (SCHO). **(b)** Map of the number of public COVID-19 test sites per county in California participating in SARS-CoV-2 genomic surveillance. In August 2022, there were 142 test sites across 43 counties in California. These sites provided COVID-19 testing to people 2 years of age or older.

CDPH sponsored 142 COVID-19 test sites across 43 counties in California to ensure access to COVID-19 testing for all Californians (Figure 1b). These sites provided COVID-19 testing free of charge to people 2 years of age or older. In May 2022, these test sites transitioned from primarily offering laboratory-based molecular COVID-19 testing to performing mostly diagnostic, point of care COVID-19 rapid antigen tests (3). Between August 1, 2022, and December 25, 2022, test sites submitted positive antigen swabs for whole genome sequencing.

### Public health officer regions in California

A map of the 58 counties in California divided into Public Health Officer Regions (PHORs) is shown in Figure 1a with 2021 population estimates (6). The five California Health Officers Regions are Association of Bay Area Health Officials (ABAHO), Greater Sacramento Region of Health Officers (GSRHO), Rural Association of Northern California Health Officers (RANCHO), San Joaquin Valley Consortium of Health Officers (SJVCHO), and Southern California Health Officers (SCHO). The number of public test sites for each county ranged from 0 to 15 with Los Angeles County having the highest population and the most sites (Figure 1b).

### SARS-CoV-2 genomic surveillance strategy

The SARS-CoV-2 genomic surveillance goals in California were based on achieving a weekly variant detection threshold of 1% at a 95% confidence interval for each public health region (7, 8). The detection threshold goals were established based on CDC guidance and a goal to provide public health authorities with situational awareness of emerging SARS-CoV-2 variants and allow lead time to understand variant disease severity, epidemiology, and therapeutic and vaccination efficacy, prior to the variant becoming the dominant variant in a community. The sample target goals were adjusted proportionally to the population of the public health region so that no region had higher than a 4% weekly detection threshold. An additional goal was to sequence at least 2–5% of reported positive SARS CoV-2 specimens (1). The percent prevalence where a SARS-CoV-2 variant could be detected given a total number of sequences in a given time frame or the detection threshold, was calculated from the following formula where the confidence threshold was 95% and, n, was the number of sequenced specimens.

$\text{Detection Threshold}=1–{\left(1-[\text{confidence}]\right)}^{\left(1/n\right)}$ .

This method was used to determine the SARS-CoV-2 variant detection threshold in California for each week (8).

We estimated that if each test site sent two positive tests per operating day for WGS, we would achieve the statewide SARS-CoV-2 variant detection threshold goals, based upon the incidence of positive tests in California from June 2022. To compensate for regions with lower historical sampling representativeness and fewer alternative test sites to obtain samples, such as the RANCHO region, all positive antigen test specimens from each testing day were sent to the laboratory for WGS.

In addition, California developed California COVIDNet at the onset of the COVID-19 pandemic to monitor SARS-CoV-2 genomic changes in California for public health benefit (1). California COVIDNet organized by CDPH is a collaboration of public health laboratories, academic institutions, and private clinical diagnostic laboratories to generate SARS-CoV-2 genomic data in California to monitor SARS-CoV-2 over time (1). In addition to positive COVID-19 antigen tests collected at the 142 public test sites from this program for California COVIDNet, residual specimens from positive COVID-19 molecular tests were also collected throughout the state and sequenced by California COVIDNet contributing to the entirety of the California-wide SARS-CoV-2 genomic surveillance program.

### Specimen handling

Swabs from positive COVID-19 antigen tests were placed in test tubes containing DNA/RNA Shield (Zymo Research, Irvine, CA) to preserve SARS-CoV-2 RNA and inactivate the virus in the specimen (9). DNA/RNA Shield at room temperature has been approved by the FDA to be effective in preserving viral RNA from SARS-CoV-2 with a limit of detection of 250 genome equivalence copies/mL (9). Test site staff received training on how to place swabs from positive antigen tests in sealed test tubes with DNA/RNA Shield. Test site workers selected two positive tests per operating day for WGS. If there was only one specimen available, then one specimen would be sent. For test sites located in counties in RANCHO and other counties that had historical low sampling representativeness (Alpine, Amador, Calaveras, Colusa, Inyo, Mariposa, Mono, Plumas, Sierra, and Tuolumne) all specimens were submitted for WGS.

Specimens were transported via a statewide courier system within 3 days of collection to California COVIDNet partner the SARS-CoV-2 Sequencing Team at the Genome Center, University of California, Davis, to be processed for WGS. Transported specimens were placed in a cooler to keep the specimens at approximately room temperature (between 40°F/4°C and 80°F/27°C) during shipment and storage. Upon arrival, specimens were examined for leakage, reconciled with an electronic manifest and placed in 96-well racks. RNA was extracted from the DNA/RNA Shield transport buffer using the *Quick*-DNA/RNA Viral kit (Zymo Research, Irvine, CA) on a KingFisher Apex Automated Extraction System (Thermo Scientific, Waltham, MA) according to manufacturer directions with one exception; the beta-mercapthoethanol reagent was replaced with 0.5 M TCEP [Tris(2-carboxyethyl) phosphine hydrochloride]. SARS-CoV-2 RNA was quantified by RT-qPCR on an IntelliQube automated PCR instrument (LGC Biosearch, Hoddesdon, United Kingdom) using the CDC-designed primers and probes (Centers for Disease Control and Prevention, 2000). A combined N1/N2 cycle threshold (C_t_) value was determined using Fastfinder software by Ugentec (Hasseelt, Belgium). All specimens received by the laboratory that could be reconciled with the manifest and were packaged correctly were sequenced. Samples with a C_t_ between 14 and 19 were diluted 1:30. Samples with a C_t_ less than 14 were diluted 1:900. Of the positive antigen tests where sequencing was attempted, all values of C_t_ were attempted to be sequenced. Sequencing libraries were generated using the xGen SARS-CoV-2 Amplicon Panel from Integrated DNA Technologies (IDT, Coralville, IA). This involved generating cDNA using LunaScript RT Super Mix Kits from New England Biolabs (NEB, Ipswich, MA) followed by multiplexed PCR using the xGen SARS-CoV-2 Amplicon Panel 96rxn and xGen UDI 10 nt Primer Plates 1–16. The xGen NGS protocol was followed to create multiplexed short-read libraries. The xGen “Normalase” procedure was not applied. The sequencing libraries were pooled using equal volumes, and five cycles of PCR performed using the KAPA Library Amplification Primer Mix (Roche Molecular Systems, Pleasanton, CA) to generate library molecules with standard Illumina adapter sequences at both ends. Samples were sequenced using the AVITI sequencer (Element Biosciences, San Diego, CA) or an Illumina platform (NextSeq2000 or NovaSeq6000).

Quality control was performed by assembling the viral genome using default run parameters with the nf-core/ viralrecon pipeline (10). Positive and negative controls were validated as process controls. Sequencing libraries were generated a second time for potentially underperforming samples (defined as yielding less than 200,000 raw reads or assemblies with more than 10 ambiguous bases). The better performing dataset was used for further analyses. All samples with over 70% genome coverage at 10x genome read depth and less than 8,000 missing bases in the final consensus passed quality control and were released to California COVIDNet partner Theiagen Genomics (Highlands Ranch, CO), for standardized genomic analysis (1, 2). At the beginning of the program, some extracted RNA specimens were sent to other California COVIDNet affiliated laboratories for WGS. These specimens were not included in the analysis for successful WGS.

### Reporting

The CDPH Viral and Rickettsial Disease Laboratory (VRDL) linked metadata from the positive COVID-19 antigen test result with the SARS-CoV-2 sequence generated by UC Davis following protocols established by COVIDNet (1, 2). VRDL downloaded metadata associated with positive COVID-19 antigen tests generated by test sites via secure file transfer protocol. The raw SARS-CoV-2 sequence data generated by UC Davis was uploaded to the COVIDNet Database and analyzed following protocols established by California COVIDNet (1, 2). Successful sequencing was defined as samples that achieved a minimum of 83% in terms of percent reference coverage and 20x genomic read depth and assigned a SARS-CoV-2 lineage. Samples not passing these criteria were not assigned a lineage and designated as “failed sequencing.”

WGS data were submitted to the public sequencing repositories (GISAID, National Center for Biotechnology Information (NCBI), and Sequence Read Archive (SRA)) by Theiagen within 1 week after determining the SARS-CoV-2 lineage. SARS-CoV-2 lineage calls and GISAID reference numbers were reported by VRDL to the California Reportable Disease Information Exchange (CalREDIE) system via transfer of comma separated value (CSV) file format and matched with prior test data and additional patient health information per state reporting guidelines (11, 12). All COVID-19 antigen test results collected at public health test sites were reported to CalREDIE per California reporting guidelines (11, 13).

### Data

SARS-CoV-2 sequence data were accessed for this analysis from a centralized sequence repository as previously described (1, 2). At the time of analysis, this database contained 115,033 SARS-CoV-2 sequences from specimens that were collected between August 1, 2022, and December 25, 2022. The date of collection of each sample was used for analysis. Samples were classified as CDPH test site samples if their associated unique barcode number was identified as originating from a CDPH sponsored test site via an internal database. Statewide COVID-19 test positivity data were sourced from the CDPH Health and Human Services Open Data Portal (14). Tests reported to CDPH Health and Human Services Open Data Portal included all laboratory-based COVID-19 diagnostic tests, and nearly all of these were molecular based (12, 14).

## Results

### COVID-19 antigen tests performed at California COVID-19 public test sites

**Figure 2:**
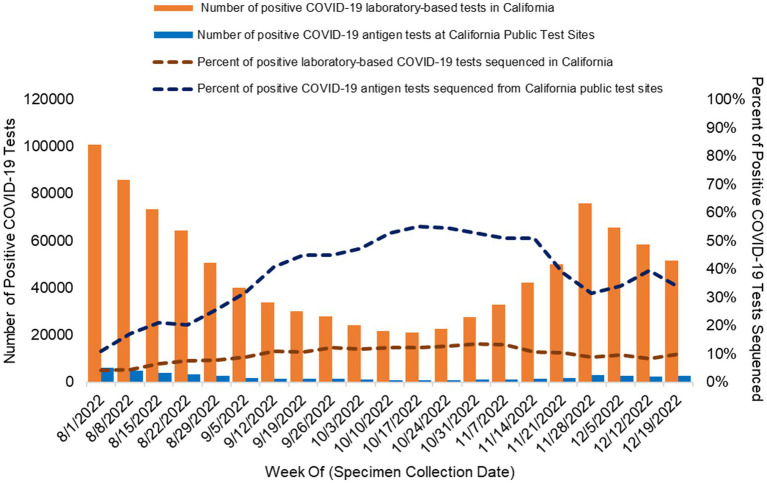
Percent of positive COVID-19 rapid antigen tests sourced from California public COVID-19 test sites sequenced and assigned to a lineage and percent of positive laboratory-based COVID-19 tests sequenced and assigned to a lineage from all sources in California. Orange bars: number of positive COVID-19 laboratory-based tests reported to CALREDIE per week statewide. Blue bars: Number of positive COVID-19 antigen tests collected at public test sites per week. The dashed lines represent the percentage of positive COVID-19 tests assigned to a lineage via WGS in California. Orange dashed line: percentage of all positive laboratory-based COVID-19 sequenced statewide each week. Blue dashed line: percentage of positive COVID-19 antigen tests sequenced from California public COVID-19 test sites each week.

Between August 1, 2022 and December 25, 2022, at 142 California COVID-19 public test sites (Figure 1b), there were 46,048 positive COVID-19 antigen tests, out of the 217,092 tests performed, and test positivity was 21.4% (46,408 / 217,092). There was a median of 1,648 positive COVID-19 antigen tests per week (range 809–6,089) at COVID-19 test sites (Figure 2). The number of positive tests collected weekly varied during the study period (Figure 2).

### WGS of positive COVID-19 antigen tests sourced from COVID-19 public test sites

There were 14,088 SARS-CoV-2 whole genome sequences generated from residual specimens from positive COVID-19 antigen tests collected at public test sites (Figure 1b) with a median of 636 sequences per week (weekly range 427–901). Overall 13.9% (14,088/101,237) of all SARS-CoV-2 sequences in California during the study period were sourced from California public COVID-19 test sites (Table 1). Sequences from California public COIVD-19 test sites made up 2.7% (14,088/513,763) of the SARS CoV-2 sequences that were reported in the United States to GISAID during the study period.

**Table 1:** SARS-CoV-2 whole genome sequencing at California public test sites and statewide.

Region	Public test sites: sequences per 100,000 per week (average & range)	All sources: sequences per 100,000 per week (average & range)	% of WGS from public test sites during study period
California statewide	1.7 (1.1–2.3)	10.6 (6.6–15.6)	13.9% (14,088/101,237)
Southern California (SCHO)	1.5 (0.9–2.3)	10.3 (5.2–17.2)	14.3% (7,165/49,946)
Bay Area (ABAHO)	1.5 (1.0–2.0)	12.2 (10.1–14.7)	12.3% (2,676/21,702)
Greater Sacramento (GSRHO)	1.5 (1.0–2.2)	9.5 (7.1–14.0)	15.5% (915/5,908)
San Joaquin Valley (SJVCHO)	2.6 (1.5–3.6)	9.8 (5.1–14.1)	25.8% (2,400/9,291)
Rural Northern California (RANCHO)	5.3 (3.4–7.4)	7.6 (5.3–10.8)	69.0% (784/1137)

In total, 30.4% (14,088/46,408) of positive COVID-19 antigen tests collected at California public test sites were associated with a reported SARS-CoV-2 sequence [weekly range 11.0% (671/6,089)–55.1% (457/830); Figure 2]. By program design, the percentage of positive COVID-19 antigen tests that underwent WGS increased when the number of positive COVID-19 antigen tests decreased in order for the weekly sequencing volume to be sufficient to meet detection threshold goals (Figure 2).

**Figure 3:**
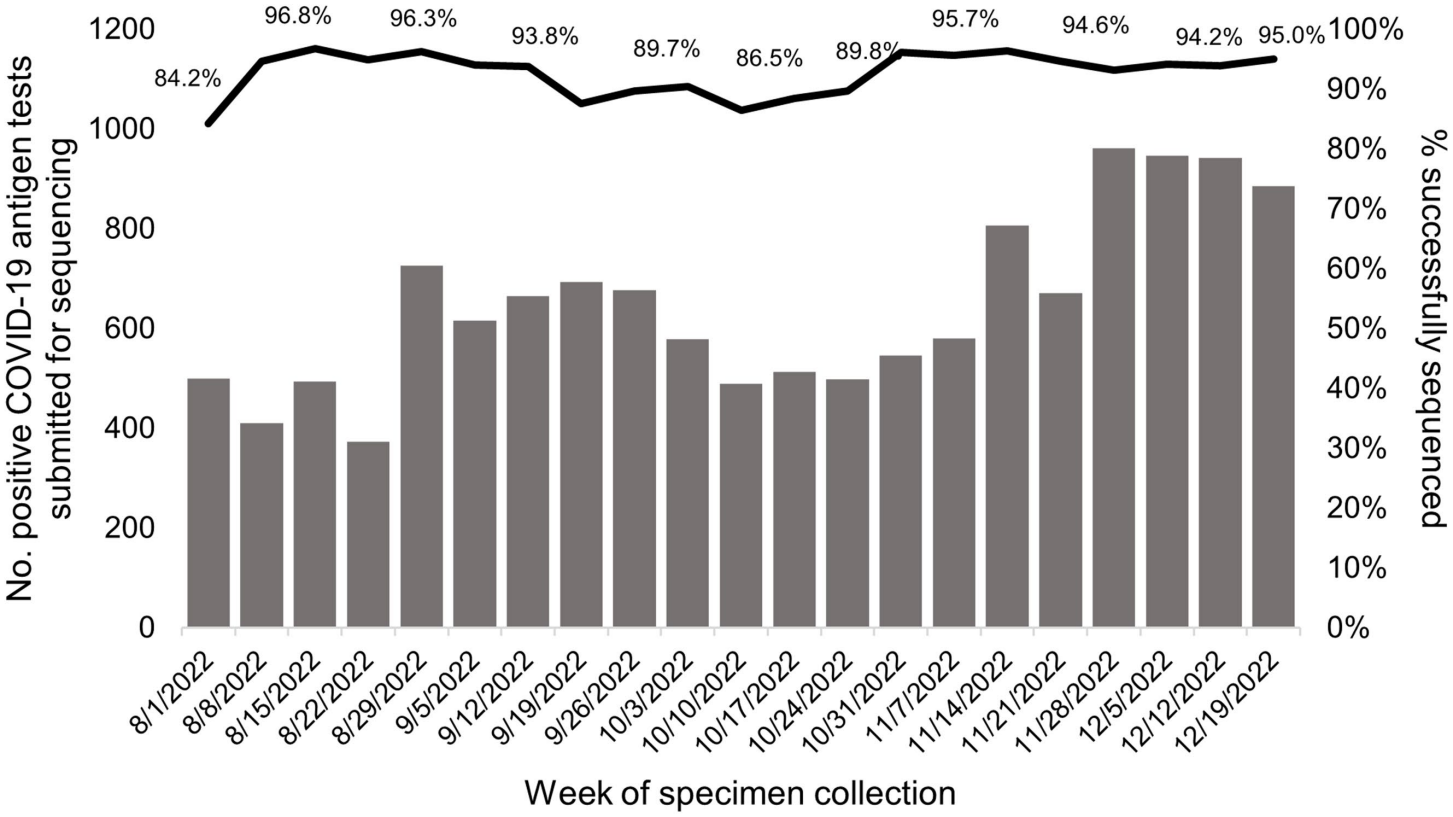
Successful SARS-CoV-2 sequencing and lineage assignment from positive COVID-19 antigen tests conducted at California Public COVID-19 test sites. The gray bars show the number of positive COVID-19 antigen tests submitted for WGS each week of the study period. The black line represents the percent of positive COVID-19 antigen tests that generated a lineage call among tests where sequencing was attempted per week. Specimens were sourced from positive COVID-19 antigen tests collected at California public COVID-19 test sites.

In total, 92.9% [12,625/13,585; weekly range 84.2% (421/500)–96.8% (478/494)] of all samples that underwent sequencing at the UC Davis genome center were successfully assigned to a SARS-CoV-2 lineage (Figure 3). WGS of positive laboratory-based SARS-CoV-2 tests

During the period of program operations, WGS of SARS CoV-2 was also occurring statewide from various testing sources (as described in Methods). During this period, there were 1,000,530 positive COVID-19 laboratory-based tests reported and a total of 87,249 SARS-CoV-2 sequences reported in California. On average, there were 47,644 positive COVID-19 laboratory tests reported each week (Range 21,045–100,806) and 4,150 SARS-CoV-2 sequences reported weekly (Range 2,595–6,805). On average, 9.8% of positive laboratory-based COVID-19 tests were sequenced weekly (range 4.1–13.6%; Figure 2).

### SARS-CoV-2 genomic surveillance august 1, 2022 to December 25, 2022

**Figure 4:**
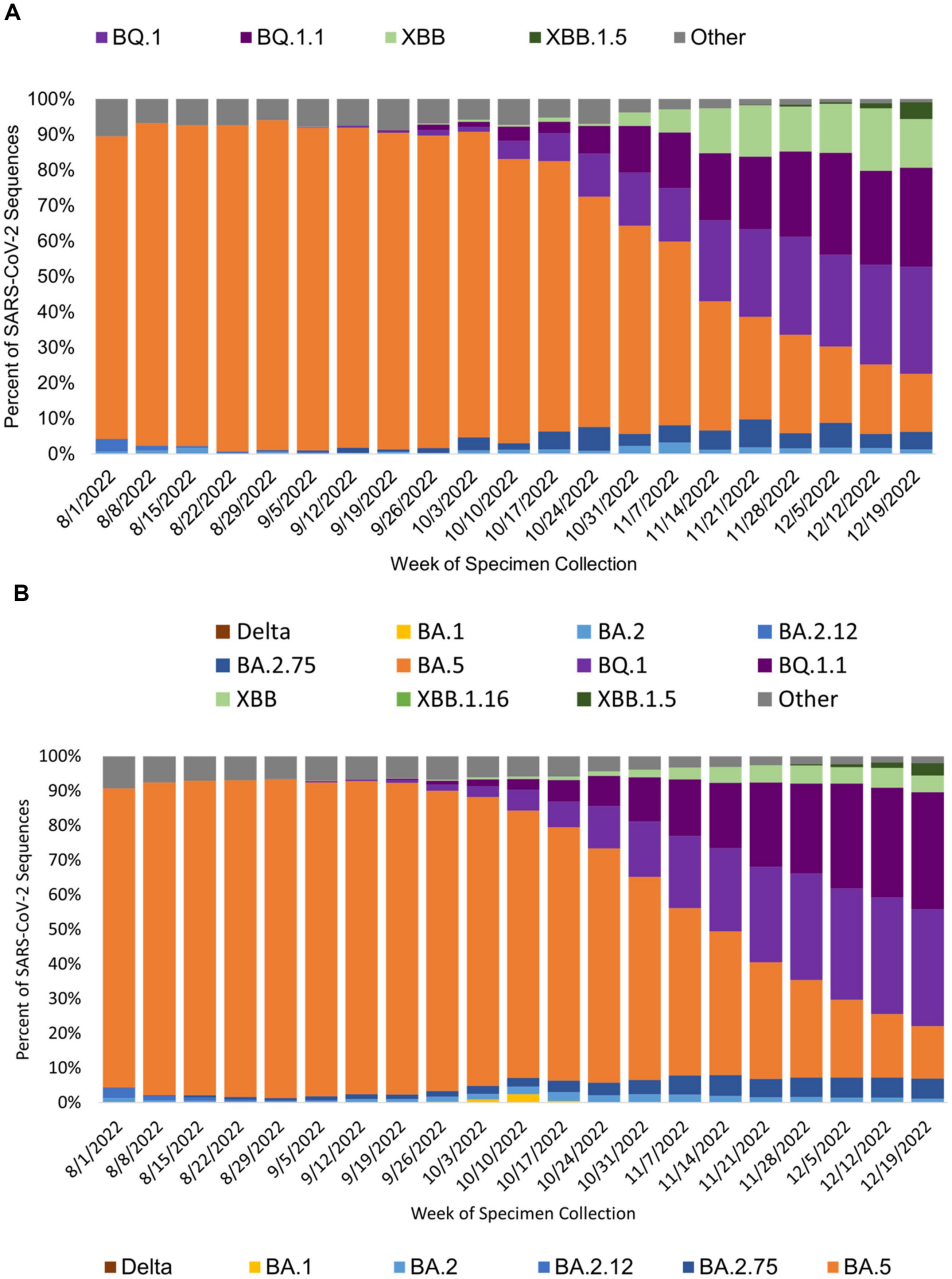
SARS-CoV-2 variant prevalence from specimens collected at **(A)** public COVID-19 test sites and **(B)** for all of California by specimen collection week. Figure shows the prevalence of SARS-COV-2 variants by week of specimen collection for sequences sourced from public COVID-19 tests sites **(A)** and all sources in California **(B)**. BA.5 was the prevalent SARS-CoV-2 during the study period.

The genomic surveillance of SARS-CoV-2 variants over the course of the study varied similarly for specimens generated from public test sites and specimens from all sources in California (Figures 4A,B). BA.5 was the most common SARS-CoV-2 variant identified during the study period, and the BQ variants were rising toward the end of the study.

### SARS-CoV-2 WGS from public test sites across California public health officer regions

**Figure 5:**
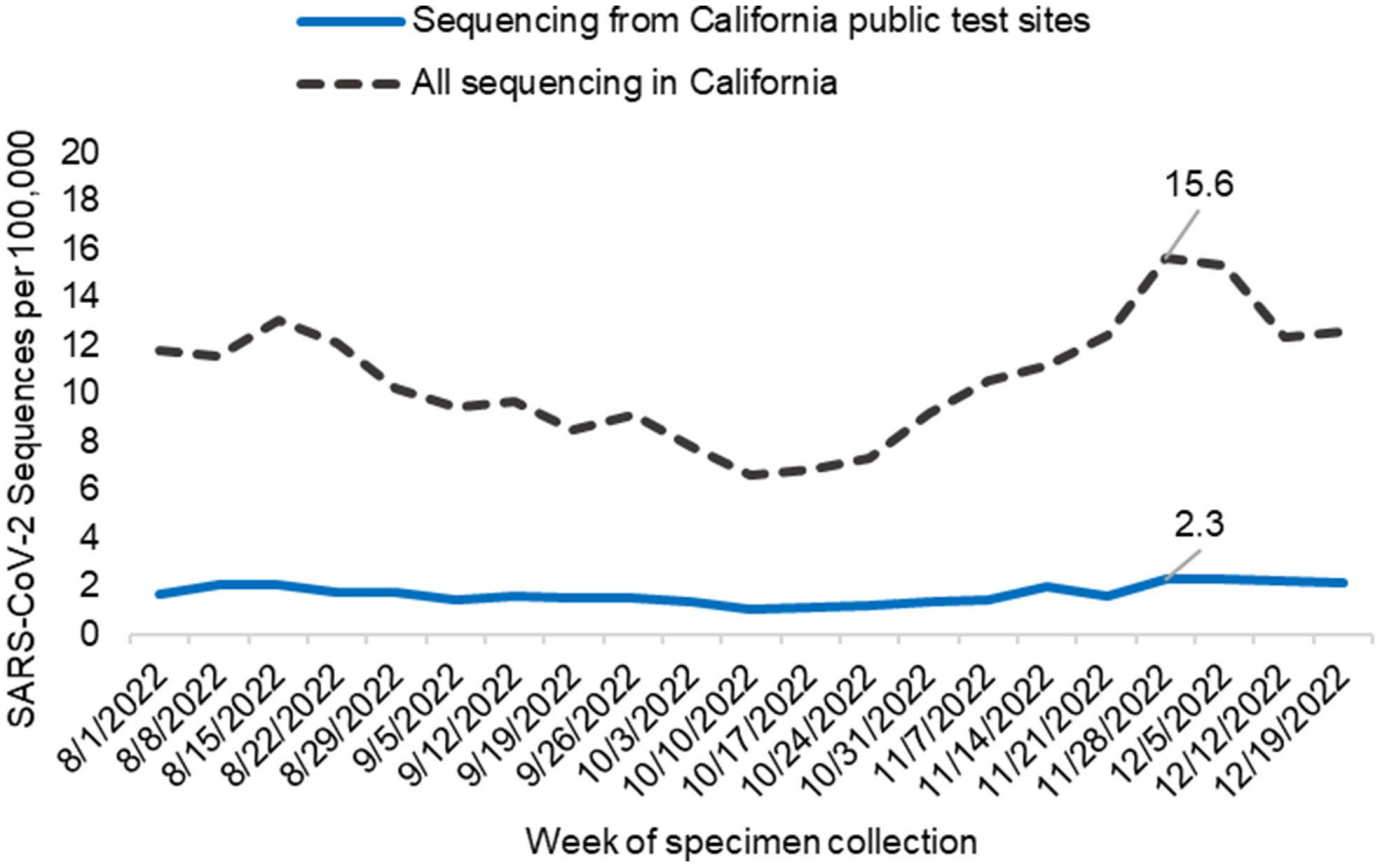
SARS-CoV-2 sequencing from public COVID-19 test sites across California. The graph indicates the number of sequences generated per 100,000 people from samples collected at public test sites (blue line) compared to all California sequencing (black hashed line) each week. Data includes only sequences that were assigned to a SARS-CoV-2 lineage.

The percentage of all SARS-CoV-2 sequences collected at California’s public COVID-19 test sites during the study period varied by public health officer region. For the RANCHO region, public COVID-19 test sites contributed 69.0% of all SARS-CoV-2 sequences from this region. SARS-CoV-2 sequences from public COVID-19 test sites from the remaining public health officer regions are as follows: SJVCHO: 25.8%; ABAHO: 12.3%; SCHO: 14.3%; GSRHO: 15.5%. Across California, on average, 10.6 sequences per 100,000 people (range 6.6–15.6) were generated each week from all sources. Of those, 1.7 sequences per 100,000 people (range 1.1–2.3) were generated each week from public COVID-19 test site samples. The average number of sequences sourced from public test sites each week per 100,000 individuals, as compared to all test sources, are summarized in Table 1 and Figure 5. The regions with the highest proportion of their specimens originating from public test sites were San Joaquin Valley region (2.6 sequences / 100,000 people, range 1.5–3.6) and RANCHO region (5.3 sequences / 100,000, range 3.4–7.4; Table 1; Supplementary Figure 1).

### Weekly SARS-CoV-2 detection thresholds across California

Statewide, from August 1, 2022, through December 25, 2022, the average weekly detection threshold for novel SARS-CoV-2 variants was 0.07% prevalence at 95% confidence (range: 0.05–0.10% weekly). The average weekly detection threshold would have increased to 0.09% (range 0.06–0.13%) if samples from public COVID-19 tests sites were not available (Table 2). The detection threshold would have been 0.43% if samples were only sourced from public test sites. This program had the biggest impact in the RANCHO region where it improved the average weekly detection threshold from 18% (range: 11–39%) to 5.2% (range: 2.8–7.1%), due to the contribution of samples collected at public COVID-19 test sites (Table 2).

**Table 2:** Average weekly novel SARS-CoV-2 detection thresholds across public health officer regions from August 1, 2022, to December 25, 2022.

Region	Average weekly detection threshold (%) at 95% confidence with all available sequences including public test site sequences (%, weekly range)	Average weekly detection threshold (%) at 95% confidence excluding public test site sequences (%, weekly range)	Average weekly detection threshold (%) at 95% confidence including only public test site sequences (%, weekly range)
Statewide	0.07% (0.05–0.10)	0.09% (0.06–0.13)	0.43% (0.24–0.62)
Southern California (SCHO)	0.14% (0.07–0.24)	0.16% (0.09–0.33)	0.82% (0.49–1.19)
Bay Area (ABAHO)	0.27% (0.23–0.33)	0.31% (0.26–0.40)	2.25% (1.24–3.35)
Greater Sacramento (GSRO)	1.0% (0.65–1.3)	1.2% (0.74–1.6)	6.37% (3.31–9.81)
San Joaquin Valley (SJVCHO)	0.68% (0.45–1.1)	0.93% (0.45–1.1%)	2.56% (1.29–4.08)
Rural Northern California (RANCHO)	5.2% (2.8–7.1)	18% (11–39)	7.36% (3.50–10.9)

### Turnaround time

The median turnaround time between collection date at the public COVID-19 test sites and the date that SARS-CoV-2 lineage data was reported to CALREDIE was 22 days (interquartile range 18–29 days).

### Costs

The cost for SARS-CoV-2 sequencing for the sequencing laboratory was approximately $120/sequence. Per specimen sequencing costs decreased with increasing sequencing volume. Cost to transport specimens was approximately $500 per week. Average sequencing cost per week was approximately $81,000. Library preparation was the largest portion of the sequencing cost ($85/specimen).

## Discussion

From August 1, 2022, through December 25, 2022, California COVIDNet sourced specimens from positive COVID-19 antigen tests from California’s COVID-19 public test sites to generate 14,088 SARS-CoV-2 sequences, which represented 13.9% of California’s SARS-CoV-2 sequences and 2.7% of all sequences in the United States during this time period. We demonstrated success in the collection, transport, and sequencing of positive COVID-19 rapid antigen tests which resulted in 93% of sequences being assigned to a SARS-CoV-2 lineage. To our knowledge, this was the first large scale implementation of SARS-CoV-2 genomic surveillance from rapid antigen tests from across a large and diverse geographical region. We leveraged lessons from our previous work using antigen tests for SARS-CoV-2 genomic surveillance from arriving international travelers at select California’s airports (manuscript in preparation). Others have demonstrated effectiveness of WGS from COVID-19 antigen tests, but at a smaller scale and not for population-based genomic surveillance (15, 16).

WGS from rapid COVID-19 antigen tests performed at 142 public test sites across California provided a statewide community-based sample source for situational awareness of circulating variants of SARS-CoV-2 and allowed California to achieve genomic surveillance goals as COVID-19 testing modalities transitioned from laboratory-based molecular testing to rapid antigen testing (Figure 1b). With public test sites included as part of the state’s SARS-CoV-2 genomic surveillance program (California COVIDNet), we achieved the goals of: (1) sequencing at least 5% of reported positive SARS CoV-2 tests (Figure 2); and, (2) establishing a detection threshold of 1% or less for SARS-CoV-2 novel variant detection in all public health officer regions, except the Rural Northern California (RANCHO) region (Table 2) (1).

Genomic surveillance from California public COVID-19 test sites was particularly important in rural, remote, regions of the state where medical services were more limited and transporting samples to a sequencing laboratory was difficult relative to non-rural regions. This community-based genomic surveillance program produced 69 and 26% of SARS-CoV-2 sequences from the rural northern region (RANCHO) and the rural central region (San Joaquin Valley) of the state, respectively (Table 1). The simplified logistics of sending positive samples directly from the test site to the sequencing laboratory benefited these rural regions. This program maintained the weekly detection threshold for new variants below 10% variant prevalence in the RANCHO region for a given week. Without this program, the detection threshold for new variants would have risen to as high as 39% variant prevalence (Table 2).

There were several advantages of using antigen tests that met public health needs for California. Antigen tests can be performed at the point of care, provide results in 15 min, and improve access to COVID-19 testing in rural areas where laboratory capacity was less and transporting specimens to a laboratory was logistically more challenging than in non-rural regions. Utilizing positive antigen test for WGS as we did here allowed for a more efficient and rapid means to obtain SARS-CoV-2 specimens for genomic surveillance and leveraged community-based sites in rural areas to improve situational awareness of circulating virus, particularly in less represented regions. WGS on the original positive antigen specimens saves time, resources, and likely increases the chance of obtaining the sequence, provided that DNA/RNA Shield or similar molecular transport medium is used to preserve the integrity of the viral RNA. As testing transitioned from laboratory-based molecular testing to rapid point of care antigen-based testing, WGS from positive COVID-19 antigen tests provided a necessary pathway to maintain SARS CoV-2 genomic surveillance. This strategy serves as a successful model for genomic surveillance for other respiratory pathogens of public health significance such as influenza and respiratory syncytial virus.

The median turnaround time (TAT) for SARS-CoV-2 sequencing from antigen tests was 22 days. This was shorter, on average, than WGS from laboratory-based molecular tests by some California COVIDNet partners (unpublished data). In contrast, processing of SARS CoV-2 molecular specimens for WGS involves more steps, including shipment of the specimen to a diagnostic testing laboratory and then in most cases to a sequencing laboratory, which results in longer turnaround times than antigen tests. This turnaround time is far longer than the 24–48 h achieved by COG-UK SARS-CoV-2 genomic surveillance program with a decentralized infrastructure and sample collection and sequencing taking place at multiple sites within a network of regional sequencing centers (17). International organizations recommend turnaround times less than 21 days to maintain SARS-CoV-2 variant situational awareness (7).

There are several limitations to this work. For one, turnaround time, was a limitation and the median was more than 21 days. However, turnaround times for this program were faster than other sequencing strategies in California. In the rural Northern California region, SARS-CoV-2 variant detection thresholds were markedly lowered by the program, however, the goal variant detection threshold of 4% was still not achieved in this region. The costs to conduct sequencing at a level to achieve the recommended detection thresholds were high. Expenses incurred were associated with the transport of specimens to the laboratory on a near-daily basis as well as with laboratory procedures including nucleic acid extraction, sequencing library preparation, sequencing, and data analysis and management. The library preparation for sequencing was the most expensive step in this process, but we expect this cost to decrease over time with the advent of high-throughput automation.

This work highlights the impact of a SARS-CoV-2 genomic surveillance program that directly sourced specimens from a statewide public COVID-19 rapid antigen testing program. The ability to use antigen tests for statewide genomic surveillance leveraged the public health benefits of antigen testing while providing actionable genomic data that benefited all of California. The California Testing Taskforce COVID-19 testing program was invaluable to ensure SARS-COV-2 testing and genomic surveillance data were available throughout the state, and particularly in rural communities where medical services can be limited. The strategy we used here to leverage community-based antigen test sites as sources for WGS samples enabled us to establish a successful, timely, and representative genomic surveillance program.

As we move beyond COVID-19, we will likely encounter another pandemic. Community-based test sites utilizing point of care rapid diagnostic tests linked to a sequencing laboratory network coupled with an integrated genomic surveillance program provide immense public health benefits during a pandemic response. With this work we showed that (1) California was successful in transitioning its SARS-CoV-2 genomic surveillance program from laboratory-based molecular tests to positive antigen tests collected at community-based test sites between August 1, 2022, through December 25, 2022 while meeting nearly all SARS-CoV-2 genomic sequencing goals; (2) community-based point of care COVID-19 testing integrated with genomic surveillance helped California maintain its SARS-CoV-2 genomic surveillance program across the state and particularly rural communities; and, (3) a strategy coupling community-based testing with a comprehensive SARS-CoV-2 genomic surveillance program serves as a model for other pathogens of public health significance.

## Data Availability

The original contributions presented in the study are included in the article/Supplementary material, further inquiries can be directed to the corresponding author.

## Supplementary material

SUPPLEMENTARY FIGURE 1 SARS-CoV-2 sequencing from public COVID-19 test sites across California Public Health Officer Regions. Graphs indicate the number of SARS-CoV-2 sequences per 100,000 people from samples collected at public test sites (blue) and collected from all sources across the region (black) each week. From top to bottom: **(a)** Southern California Health Officers (SCHO); **(b)** Association of Bay Area Health Officials (ABAHO); **(c)** Greater Sacramento Region of Health Officers (GSRHO); **(d)** San Joaquin Valley Consortium of Health Officers (SJVCHO); **(e)** Rural Association of Northern California Health Officers (RANCHO).
