# Supplementary figures and images for: SARS-CoV-2 genomic surveillance using rapid point of care COVID-19 antigen tests at public test sites in California
Source: Front Public Health. 2025 Jul 25;13:1620651. doi: 10.3389/fpubh.2025.1620651 (PMC12331611; doi:10.3389/fpubh.2025.1620651)

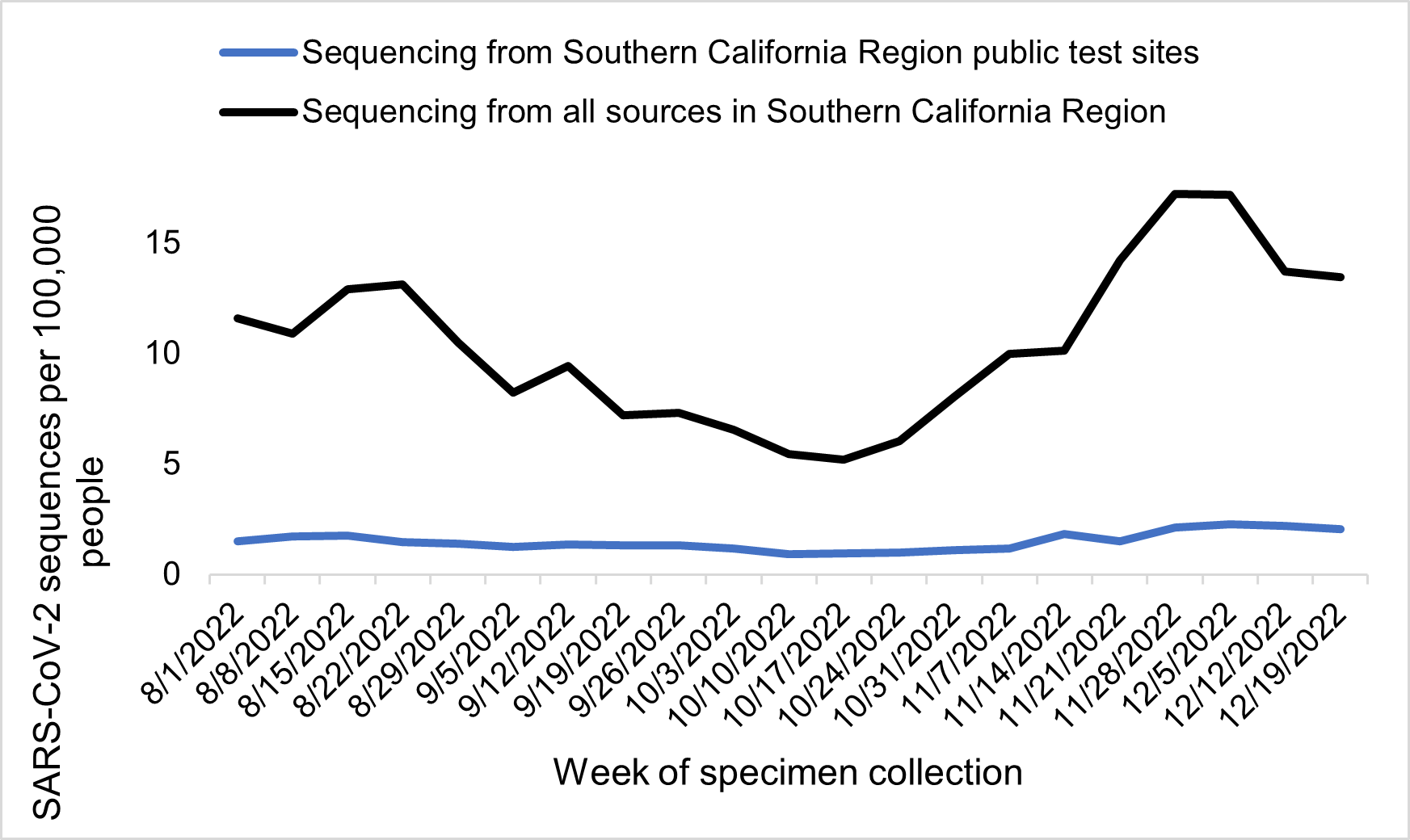

Supplement: SUPPLEMENTARY FIGURE 1 — SARS-CoV-2 sequencing from public COVID-19 test sites across California Public Health Officer Regions. Graphs indicate the number of SARS-CoV-2 sequences per 100,000 people from samples collected at public test sites (blue) and collected from all sources across the region (black) each week. From top to bottom: (a) Southern California Health Officers (SCHO); (b) Association of Bay Area Health Officials (ABAHO); (c) Greater Sacramento Region of Health Officers (GSRHO); (d) San Joaquin Valley Consortium of Health Officers (SJVCHO); (e) Rural Association of Northern California Health Officers (RANCHO). [file Image_1.tif]

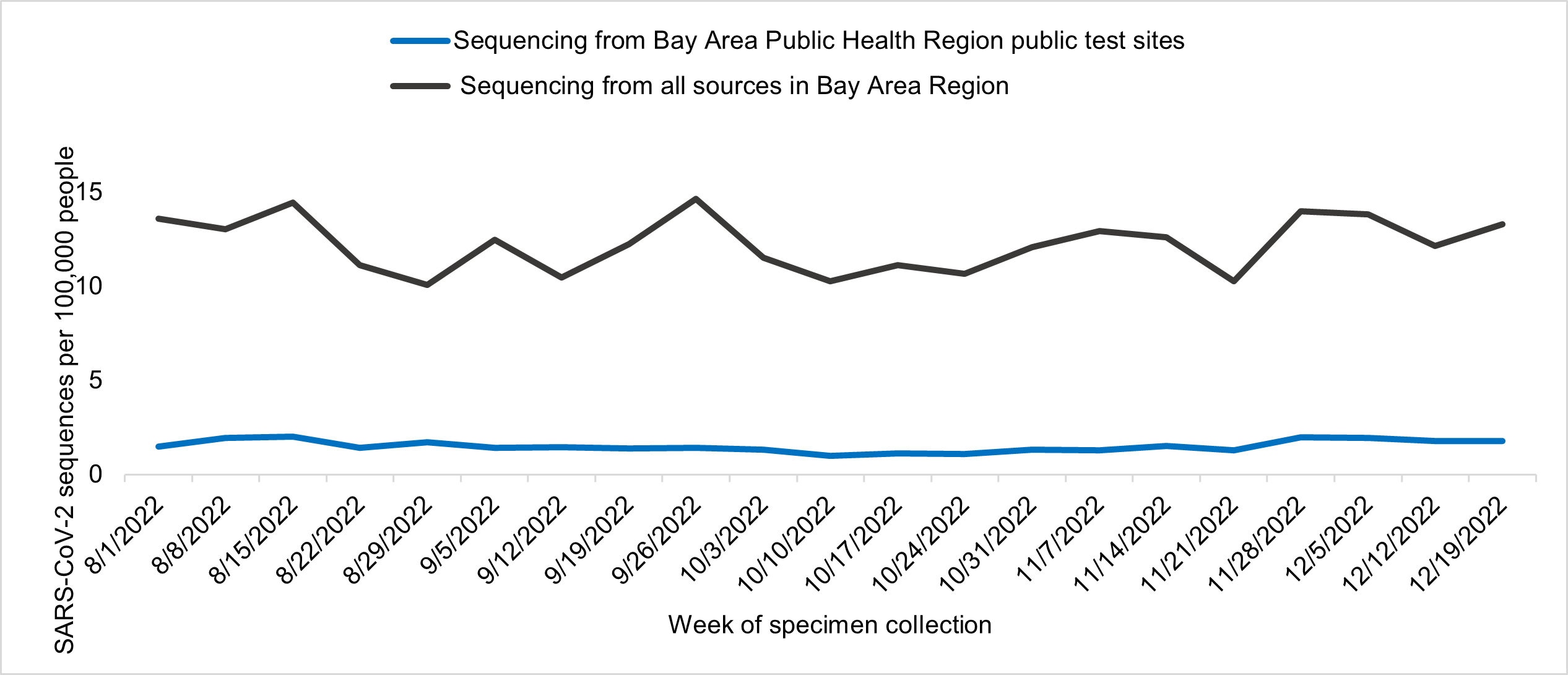

Supplement: Supplementary file 2 [file Image_2.tif]

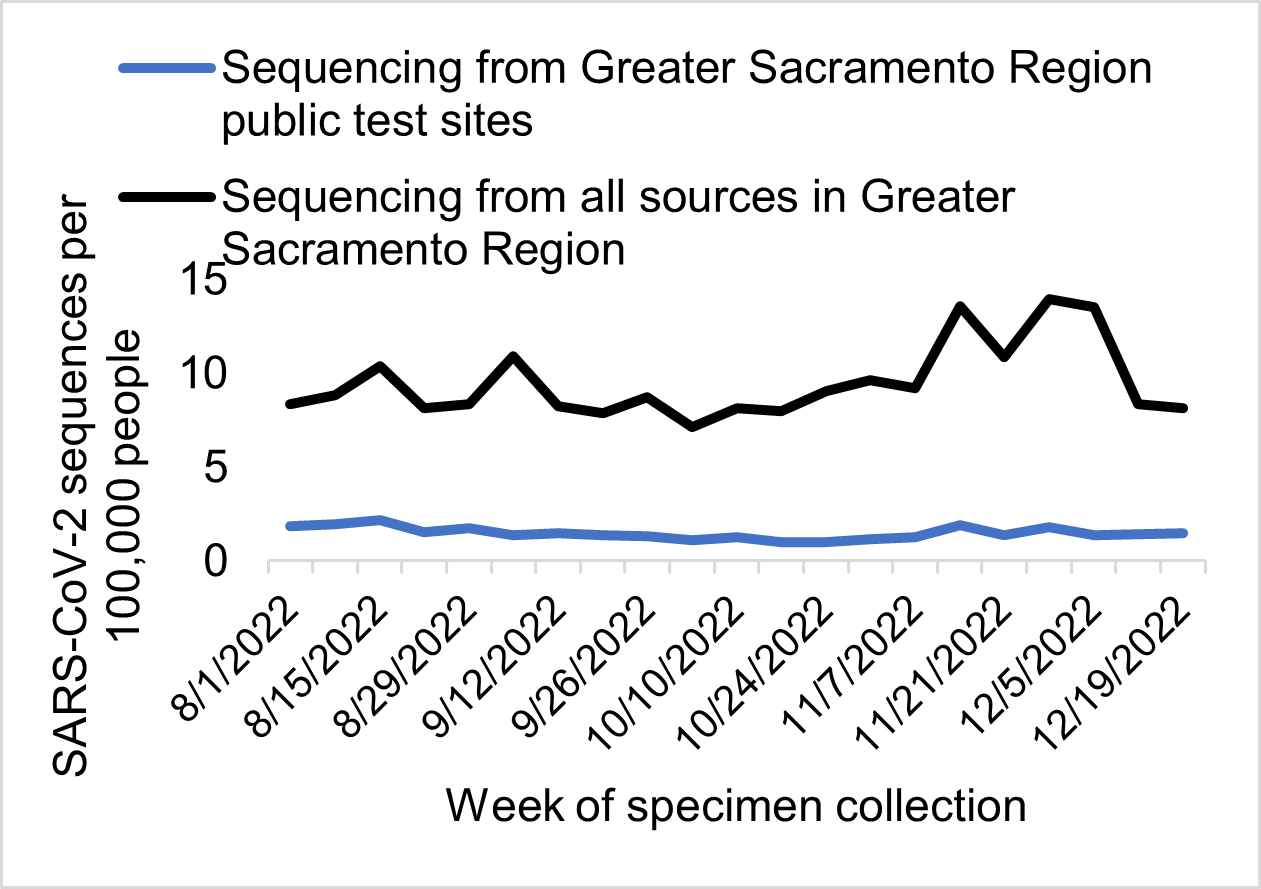

Supplement: Supplementary file 3 [file Image_3.tif]

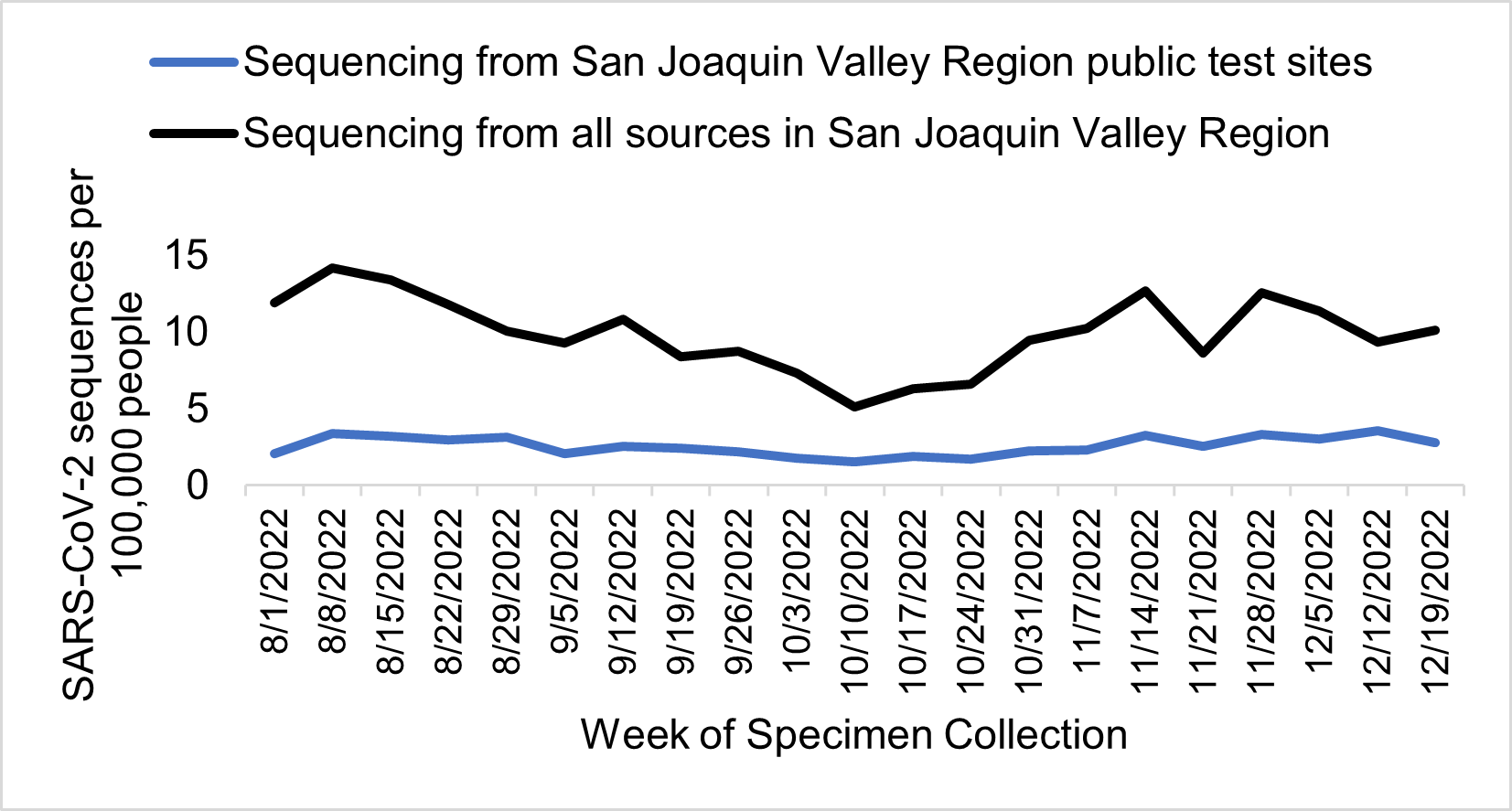

Supplement: Supplementary file 4 [file Image_4.tif]

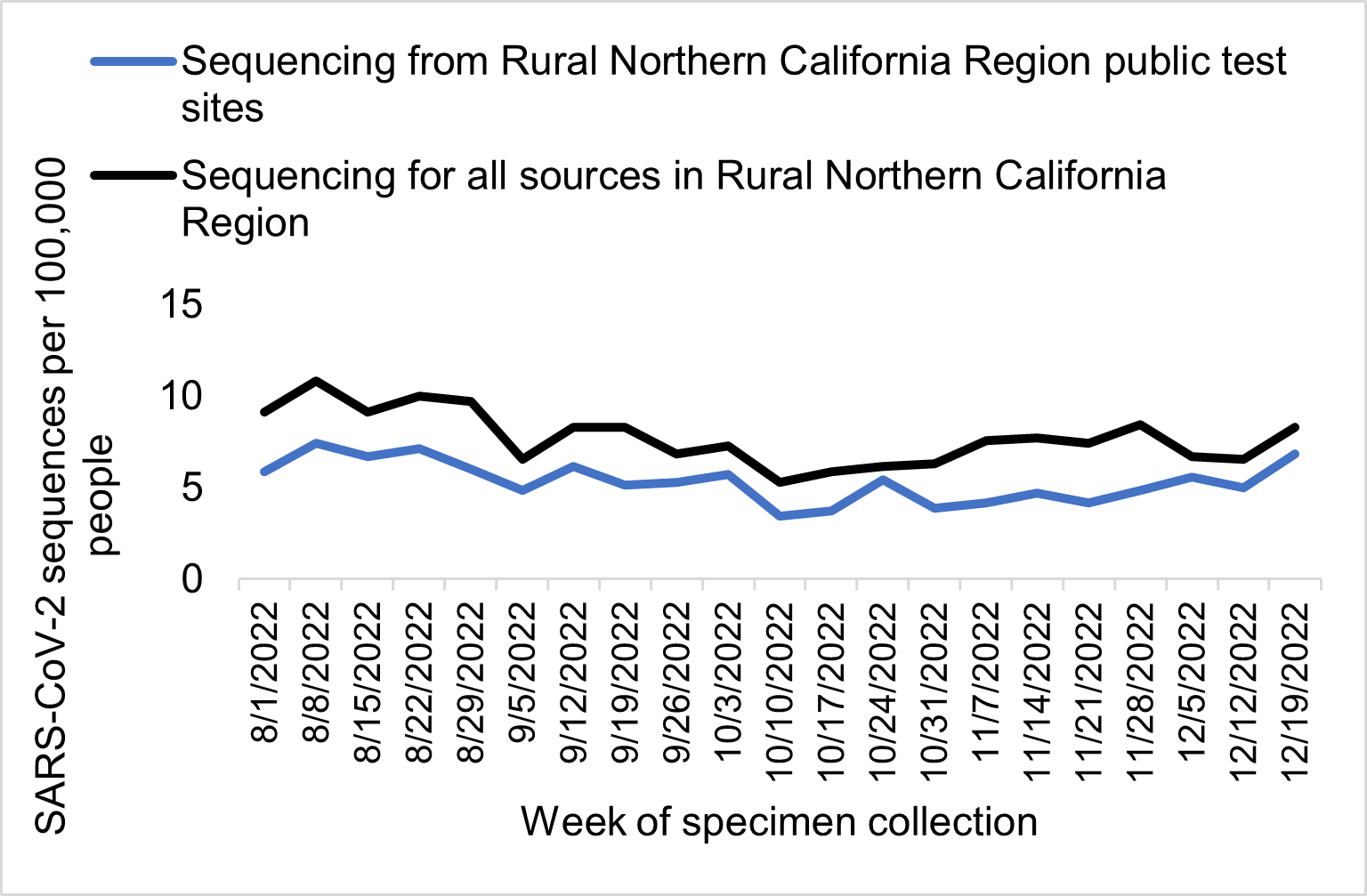

Supplement: Supplementary file 5 [file Image_5.tif]
